# Efficiency aspects of regioselective testosterone hydroxylation with highly active CYP450‐based whole‐cell biocatalysts

**DOI:** 10.1111/1751-7915.14378

**Published:** 2023-11-29

**Authors:** Carolin Bertelmann, Magdalena Mock, Andreas Schmid, Bruno Bühler

**Affiliations:** ^1^ Department of Solar Materials Leipzig Germany; ^2^ Department of Microbial Biotechnology Helmholtz Centre for Environmental Research GmbH–UFZ Leipzig Germany; ^3^ Present address: Department of Mechanical Engineering and Material Sciences Georg Agricola University of Applied Sciences Bochum Germany

## Abstract

Steroid hydroxylations belong to the industrially most relevant reactions catalysed by cytochrome P450 monooxygenases (CYP450s) due to the pharmacological relevance of hydroxylated derivatives. The implementation of respective bioprocesses at an industrial scale still suffers from several limitations commonly found in CYP450 catalysis, that is low turnover rates, enzyme instability, inhibition and toxicity related to the substrate(s) and/or product(s). Recently, we achieved a new level of steroid hydroxylation rates by introducing highly active testosterone‐hydroxylating CYP450 BM3 variants together with the hydrophobic outer membrane protein AlkL into *Escherichia coli*‐based whole‐cell biocatalysts. However, the activity tended to decrease, which possibly impedes overall productivities and final product titres. In this study, a considerable instability was confirmed and subject to a systematic investigation regarding possible causes. In‐depth evaluation of whole‐cell biocatalyst kinetics and stability revealed a limitation in substrate availability due to poor testosterone solubility as well as inhibition by the main product 15β‐hydroxytestosterone. Instability of CYP450 BM3 variants was disclosed as another critical factor, which is of general significance for CYP450‐based biocatalysis. Presented results reveal biocatalyst, reaction and process engineering strategies auguring well for industrial implementation of the developed steroid hydroxylation platform.

## Graphical abstract

Poor stability was found to critically affect testosterone (T) hydroxylation efficiency of *E. coli*‐based whole‐cell biocatalysts equipped with highly active BM3 variants and AlkL. Beside hydrophobic substrate solubility and product inhibition, inherent instability of BM3 variants was identified as most critical for T conversion into 2β‐ and 15β‐hydroxytestosterone (2/15β‐OH‐T) involving NADPH recycling via glucose catabolism.

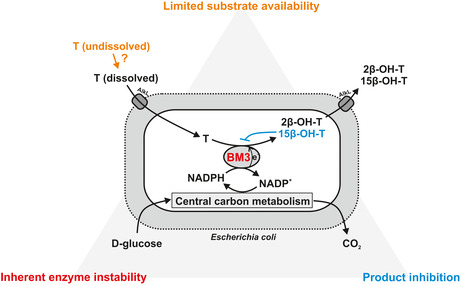

## INTRODUCTION

Cytochrome P450 monooxygenases (CYP450s) are among nature's most prevalent and versatile biocatalysts for regio‐ and stereospecific hydrocarbon oxyfunctionalization. Their ability to activate molecular oxygen and introduce one oxygen atom into inert C‐H bonds enables a wide scope of reaction types (e.g. hydroxylation, desaturation and epoxidation) (Bernhardt, 2006). This qualifies CYP450s as outstanding biocatalysts with high importance for chemically challenging industrial transformations (Guengerich & Yoshimoto, 2018; Huang & Groves, 2018; Lewis et al., 2011; Schmid et al., 2001). Steroid hydroxylation constitutes an especially relevant example, often surpassing chemical routes by selectivity and environmental friendliness or by being the only feasible option (Bureik & Bernhardt, 2007; Donova, 2017; Julsing et al., 2008). Respective hydroxylated steroid derivatives enjoy top priority for industry as they offer a wide spectrum of pharmaceutical applications, for example as contraceptives or anti‐inflammatory agents (Bureik & Bernhardt, 2007; Donova, 2017; Donova & Egorova, 2012).

Discovery of novel steroid‐hydroxylating CYP450s, as well as enzyme and strain engineering, have thus attracted high scientific interest in recent decades (Donova, 2017; Fernández‐Cabezón et al., 2018; Szaleniec et al., 2018). However, compared with other enzymes regularly used in industry such as hydrolases, practical large‐scale applications exploiting oxygenases of both eukaryotic and prokaryotic origin remain scarce (Bernhardt & Urlacher, 2014; Fernández‐Cabezón et al., 2018; Julsing et al., 2008). Major bottlenecks comprise intrinsic properties such as cofactor dependence and instability (Urlacher & Schmid, 2006; van Beilen et al., 2003). Consequently, metabolically active cells represent the preferred format for CYP450‐catalysis, enabling enzyme resynthesis and stabilization, cofactor regeneration, as well as reactive oxygen species (ROS) degradation (Duetz et al., 2001; Schrewe et al., 2013; Woodley, 2006). Still, most CYP450s exhibit low turnover rates and depend on additional redox partners delivering electrons to the active site (Bernhardt & Urlacher, 2014; Guengerich, 2002; Julsing et al., 2008; Urlacher & Eiben, 2006).

Constraints of whole‐cell biocatalyst formats include low activities and expression levels and limiting substrate uptake (Urlacher & Schmid, 2006). Recently, we tackled these issues by introducing highly active variants of the catalytically self‐sufficient CYP450 BM3 (BM3, in CYP450 nomenclature: CYP102A1) from *Bacillus megaterium* ATCC14581 (Kille et al., 2011) into living *Escherichia coli* (*E. coli*) cells (Bertelmann et al., 2022). In addition, the combination with the hydrophobic outer membrane pore AlkL was investigated. AlkL natively is associated with an alkane degradation pathway in *Pseudomonas putida* GPo1 (van Beilen et al., 1992), and its role as an alkane uptake facilitator has been demonstrated in recombinant *E. coli* (Grant et al., 2014; Julsing, Schrewe, et al., 2012). AlkL relieved uptake constraints for additional hydrophobic substrates into *E. coli*, for example fatty acid (m)ethyl esters (Julsing, Schrewe, et al., 2012; Ladkau et al., 2016; van Nuland et al., 2016), monoterpenes (Cornelissen et al., 2013) and the steroid testosterone (Bertelmann et al., 2022). Cells producing AlkL and BM3 variants showed unchanged growth characteristics and exceptionally high testosterone hydroxylation rates of up to 34 U g_CDW_^−1^. These specific activities exceeded those reported for other testosterone‐hydroxylating CYP450‐based whole‐cell biocatalysts by a factor of ≥202 (Bertelmann et al., 2022; Bracco et al., 2013), illustrating the potential to achieve high space–time yields (STYs) and steroid titres at industrial scale. However, conversion rates appeared to decrease during resting‐cell activity assays, which may be related to substrate depletion, enzyme operation and/or cell physiology (Schrewe et al., 2013). Despite their high activities and the recent extension of their substrate spectrum, engineered BM3 variants often suffer from poor coupling efficiency, that is the ratio of product formation and NAD(P)H consumption rates (Whitehouse et al., 2010). Cell physiology may be affected by high oxygenase expression and activity levels, low substrate solubility and bioavailability and/or possible inhibitory or toxic effects of substrates and products (Bernhardt & Urlacher, 2014; Urlacher & Schmid, 2006; van Beilen et al., 2003). Such issues compromise whole‐cell biocatalyst functionality in terms of specific activity and operational stability, ultimately resulting in a hampered overall efficiency of the desired biocatalytic reaction regarding final product titre and STY.

**FIGURE 1:**
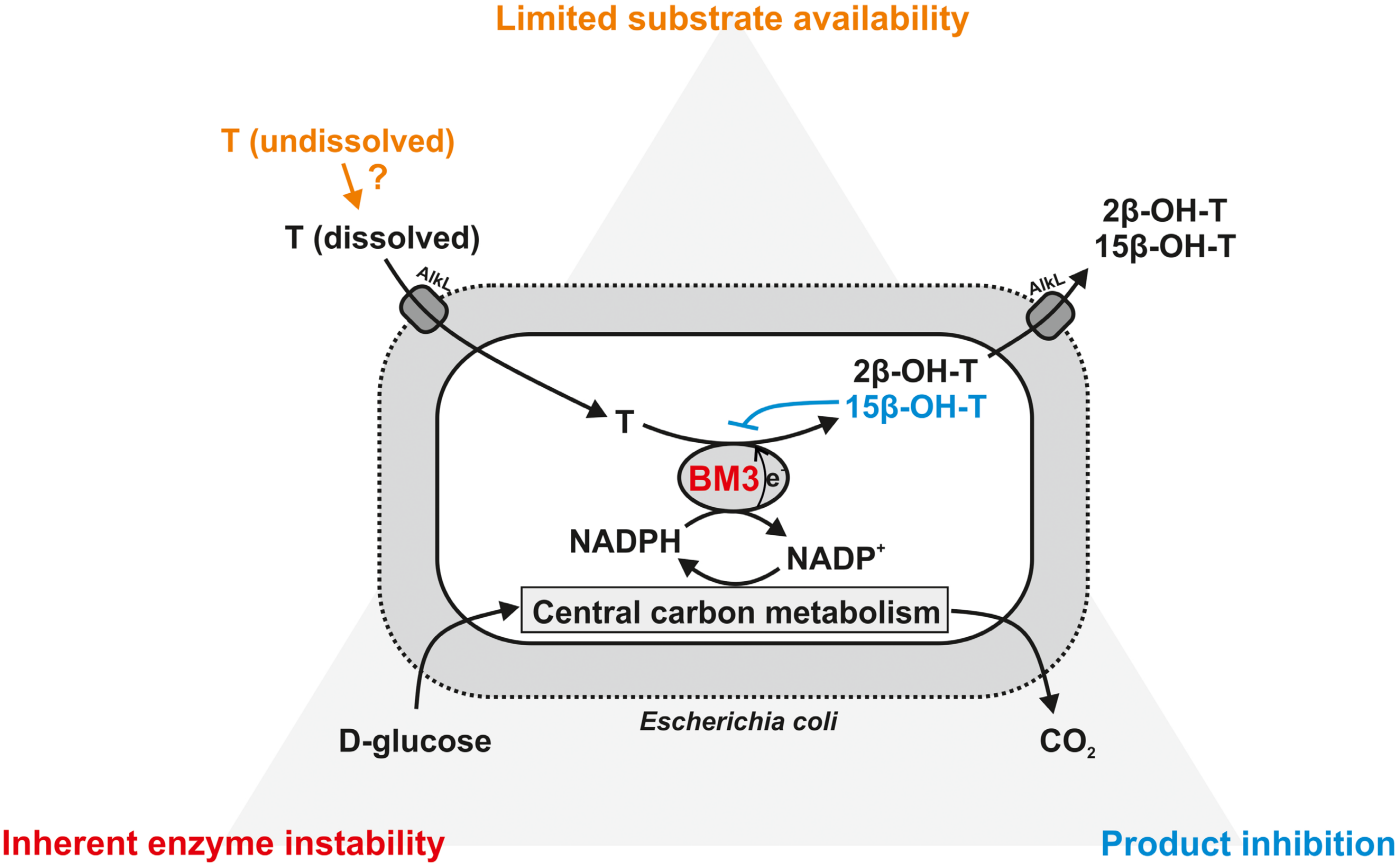
Critical aspects limiting testosterone hydroxylation efficiency of *E. coli*‐based whole‐cell biocatalysts equipped with highly active BM3 variants and AlkL. Engineered cells convert testosterone (T) into 2β‐ and 15β‐hydroxytestosterone (2/15β‐OH‐T) and recycle NADPH via glucose catabolism. Limited availability of the hydrophobic substrate T, inhibition by the main product 15β‐OH‐T and inherent instability of BM3 variants constitute possible limiting factors as they are investigated in this study.

In this study, we investigate substrate availability, enzyme instability and product inhibition as possible critical factors affecting steroid hydroxylation by highly active CYP450 (BM3)‐based whole‐cell biocatalysts (Figure 1). For this purpose, recently developed *E. coli*‐based biocatalysts equipped with highly active BM3 variants and AlkL (Bertelmann et al., 2022) were subjected to a systematic evaluation of kinetics and stability, with 2β‐ or 15β‐hydroxylation of testosterone as model reaction. Finally, we discuss different strategies to overcome the identified bottlenecks with the ultimate goal to exploit the full synthetic potential of the available whole‐cell biocatalysts.

## EXPERIMENTAL PROCEDURES

### Bacterial strains, plasmids and chemicals

*Escherichia coli* BL21‐Gold(DE3) (F^−^ *ompT hsdS(r*_*B*_^*−*^ *m*_*B*_^*−*^ *) dcm* ^*+*^ *Tet* ^*R*^ *gal*λ(DE3) *endA* Hte) was obtained from Agilent Technologies and used as recombinant host strain. As expression vector, we used the pMB1‐derived plasmid pETM11 carrying genes encoding the hydrophobic outer membrane pore AlkL and a BM3 variant (either KSA1, KSA2, KSA3, KSA14 or KSA14m; respective amino acid substitutions provided in Table S1) under control of the T7‐expression system (Bertelmann et al., 2022). 2β‐ and 15β‐hydroxytestosterone were acquired from Steraloids Inc. and Cfm Oskar Tropitzsch GmbH. All other chemicals were purchased from AppliChem, Carl Roth, Chemsolute or Sigma‐Aldrich in the highest purity available.

### Cultivation media and conditions

Bacteria were either grown in lysogeny broth (LB) medium (Sambrook & Russell, 2001) or M9 medium (Sambrook & Russell, 2001) with a pH of 7.2 containing 0.1% (v/v) US* trace element solution (Panke, Meyer, et al., 1999), 2 mM MgSO_4_ and 0.5% (w/v) D‐glucose as sole carbon and energy source. Kanamycin (50 μg mL^−1^) was added for plasmid selection. Cell growth and heterologous gene expression were carried out in baffled Erlenmeyer shake flasks with a liquid volume of maximally 20% of the total volume in a Multitron shaker (Infors). Microorganisms were incubated in a LB preculture at 37°C and 200 rpm for 6–8 h, from which a M9 preculture was inoculated (1% v/v) and incubated at 30°C and 200 rpm for another 14–16 h. This preculture was used to inoculate a M9 main culture to an optical density of 0.2 at 450 nm (OD_450_). Heterologous gene expression was induced in the early exponential phase (OD_450_ ~ 0.6) by addition of 0.1 mM isopropyl β‐D‐1‐thiogalactopyranoside (IPTG). Simultaneously, 0.5 mM of the haem precursor 5‐aminolevulinic acid (ALA) were added for enhanced haem synthesis. Incubation was continued for another 5 h followed by cell harvesting by centrifugation (5000 *g*, 5 min, 4°C) for resting‐cell biotransformations.

### Resting‐cell biotransformations

After cultivation, induction and harvesting as described above, resulting cell pellets were washed once and then resuspended in ice‐cold 100 mM potassium phosphate buffer (pH 7.4, KP_i_ buffer) supplemented with 1% (w/v) glucose to a defined cell concentration of 1 g_CDW_ L^−1^ (unless stated otherwise). Resting‐cell activity assays to investigate kinetics and biocatalyst stability were performed at a 1 mL‐scale in screw‐capped glass tubes (12 mL). Cell suspensions were adapted for 15 min in a shaking water bath at 30°C and 250 rpm. Biotransformation was started by the addition of 10 μL of a 100 mM testosterone stock solution in dimethyl sulfoxide (DMSO), leading to final concentrations of 1 mM testosterone and 1% (v/v) DMSO (unless indicated otherwise). 100 μL samples were collected after different time intervals and mixed with 12.5 μL HCl (1 M) to quench the reaction. Pure acetonitrile was added (50% v/v) to dissolve precipitated steroids, followed by mixing (2000 rpm, 5 min, 4°C) and centrifugation (17,000 *g*, 5 min, 4°C) for biomass removal. The resulting supernatant was stored at −20°C until further analysis. All assays were conducted in biological duplicates for each condition. Specific activities are given in U g_CDW_^−1^ (with 1 U defined as the activity forming 1 μmol of product per min) and were calculated based on the sum of 2β‐ and 15β‐hydroxytestosterone formed, divided by the cell concentration applied. For the different evaluations targeted in this study, the standard setup was modified as follows:

Testosterone limitation was examined by supplying a pulse of fresh substrate (1 mM) to the reaction mixture 60 min after biotransformation initialization.

Kinetic parameters for testosterone hydroxylation were determined via short‐term assays (5 min) applying testosterone concentrations ranging from 0.02 to 5 mM and a biocatalyst concentration of 0.09 g_CDW_ L^−1^. Biotransformation mixtures contained 1% (v/v) DMSO independently of the testosterone concentration applied. OriginPro 2018 software was used for curve fitting and calculation of kinetic parameters.

Mass transfer effects, especially for O_2_, were evaluated by varying the biocatalyst concentration between 0.17 and 1.9 g_CDW_ L^−1^ with a given testosterone concentration as reported before (Bertelmann et al., 2022). Optionally, 1 mM NADPH was added at biotransformation initialization.

To investigate product inhibition effects, samples of resting cells were pre‐incubated for 15 min with different concentrations of the main product 15β‐hydroxytestosterone followed by whole‐cell activity assays (5 min) initiated by testosterone addition. The final DMSO concentration was kept at 1% (v/v) in every case.

The reuse of resting cells was evaluated by harvesting cells from the reaction mixture 60 min after initial substrate addition (5000 g, 5 min, 4°C), followed by washing and re‐suspending the cells in fresh reaction buffer. Reactions were re‐started by the addition of 1 mM testosterone.

Biocatalyst stability in the biotransformation buffer under reaction conditions was studied by varying the pre‐incubation time prior to short‐term assays (5 min) initiated by testosterone addition.

Expression kinetics and biocatalyst stability in M9 medium were analysed by harvesting cells after different times of induction followed by short‐term resting cell assays (5 min).

The effect of reaction temperature was investigated by incubation at 30 and 25°C.

### Co‐solvent toxicity and inhibition studies

Toxicity: M9 cultures were prepared as described above. Simultaneously with induction, different DMSO concentrations were added to the cultures. Growth was monitored in biological duplicates at 30°C and 200 rpm until the control cultures without DMSO reached the stationary phase.

Inhibition: Resting cells were prepared and equilibrated for short‐term activity assays (5 min) as described above. Reactions were started by addition of 1 mM testosterone from differently concentrated stock solutions in DMSO for final DMSO concentrations ranging from 1% to 10% (v/v).

### Analytical methods

Suspended biomass concentrations were determined photometrically by measuring the optical density at a wavelength of 450 nm (Libra S11 spectrophotometer, Biochrom Ltd., Cambridge, UK). One OD_450_ unit corresponds to 0.166 g_CDW_ L^−1^ (Blank et al., 2008).

Monitoring of protein synthesis was carried out by harvesting 80 μg of cell dry weight (CDW) from cultures for sodium dodecyl sulfate‐polyacrylamide gel electrophoresis (SDS‐PAGE) according to Laemmli (Laemmli, 1970). Proteins extracted from 15 μg_CDW_ were loaded per lane and stained with Coomassie Brillant Blue R‐250. PageRuler™ Prestained Protein Ladder (Thermo Fisher Scientific) was used as reference.

Glucose availability in resting‐cell reaction mixtures was checked with Medi‐Test glucose test stripes (Macherey‐Nagel).

Steroid concentrations were determined via HPLC using a Dionex Ultimate 3000 system (Thermo Fisher Scientific) equipped with a Syncronis C18 column (150 x 2.1 mm, 3 μm particle size, Thermo Fisher Scientific) and an UV detector operating at 245 nm for steroid detection. Steroids (5 μL injection volume) were quantified by elution at a column oven temperature of 40°C with 55% acetonitrile in ultrapure water as a mobile phase at a flow rate of 0.5 mL min^−1^. Steroid concentrations were calculated based on peak areas and calibration curves established with commercially available standards.

## RESULTS

### The stability of high testosterone hydroxylation activities is limited

**FIGURE 2:**
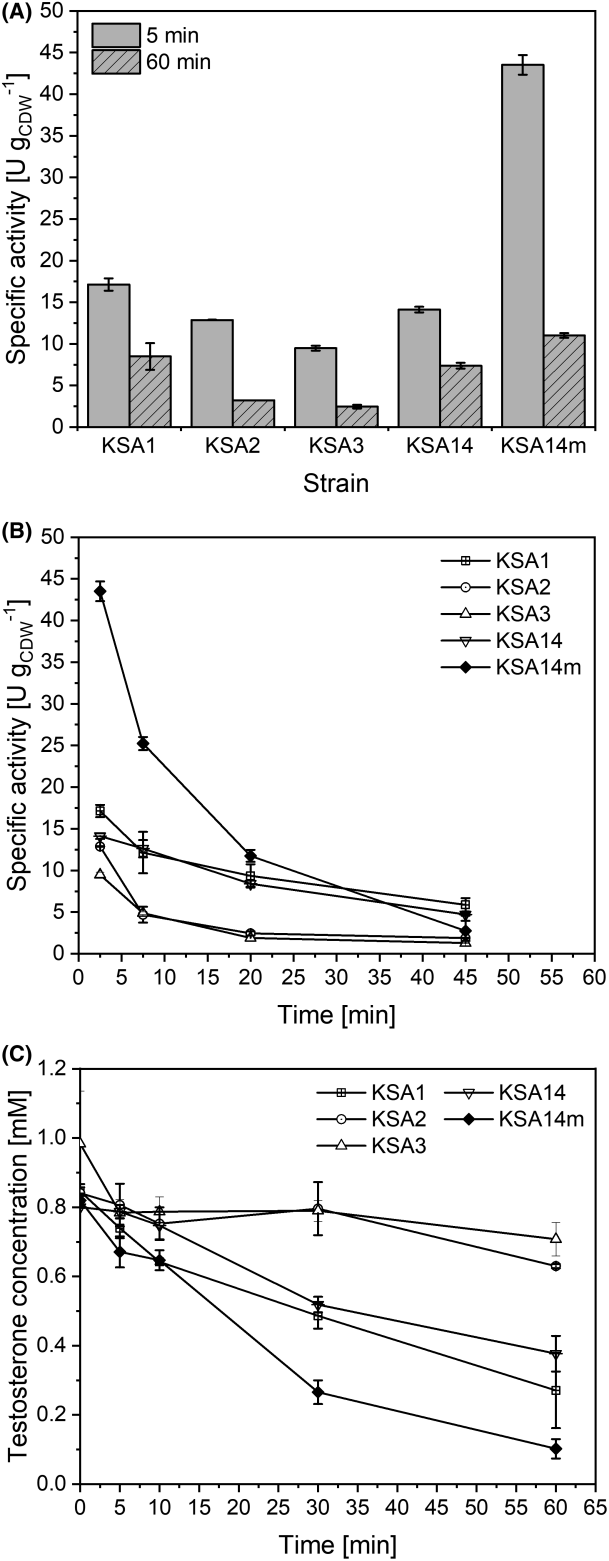
Operational stability of testosterone hydroxylation by resting *E. coli* BL21‐Gold(DE3) cells carrying pETM11 with genes encoding AlkL and different BM3 variants. Growth and heterologous gene expression were performed in M9 medium supplemented with 0.5% (w/v) glucose. Resting cells were prepared 5 h after induction with 0.1 mM IPTG and used in biotransformation assays as described in the Materials and Methods section. (A) Comparison of initial (5 min) and long‐term activities (60 min) for the respective strains. (B) Specific activity time courses. (C) Testosterone concentration time courses. Average values and standard deviations of two biological replicates are given.

In our previous study, a restrained substrate uptake proved to be a major bottleneck in *E. coli*‐based whole‐cell conversions of the steroid testosterone (Bertelmann et al., 2022). The application of suitable hydrophobic outer membrane proteins, that is hydrophobic pores, was found to relieve substrate mass transfer limitation and increased whole‐cell testosterone hydroxylation activities 6‐ to 28‐fold. Strains equipped with the membrane protein AlkL in combination with different BM3 variants eventually demonstrated high initial specific activities ranging from 9.5 ± 0.3 to 43.5 ± 1.2 U g_CDW_^−1^ depending on the applied BM3 variant, constituting a set of whole‐cell biocatalysts with the potential to improve STYs and product titres of microbial steroid conversion processes. In order to investigate the stability of these highly active biocatalysts, we set out to analyse the biotransformation time course. Specific activities calculated for 60 min of biotransformation were found to be 1.9 to 4.0 times lower than initial activities (Figure 2A). Cells showing the highest initial conversion rates displayed the fastest activity decay (Figure 2B) with the substrate concentration approaching low levels (Figure 2C) pointing to substrate limitation as a cause for the activity decrease. Catalyst instability issues due to inherent enzyme instability and/or substrate/product inhibition may however also cause or contribute to this effect. In order to investigate reasons for the rapid activity decline, we investigated whole‐cell reaction kinetics and operational stability in detail. Cells equipped with the BM3 variant KSA14m and the membrane protein AlkL were chosen as they emerged as the most active steroid‐converting whole‐cell biocatalyst (Bertelmann et al., 2022).

### Ensuring substrate availability does not relieve activity loss

**FIGURE 3:**
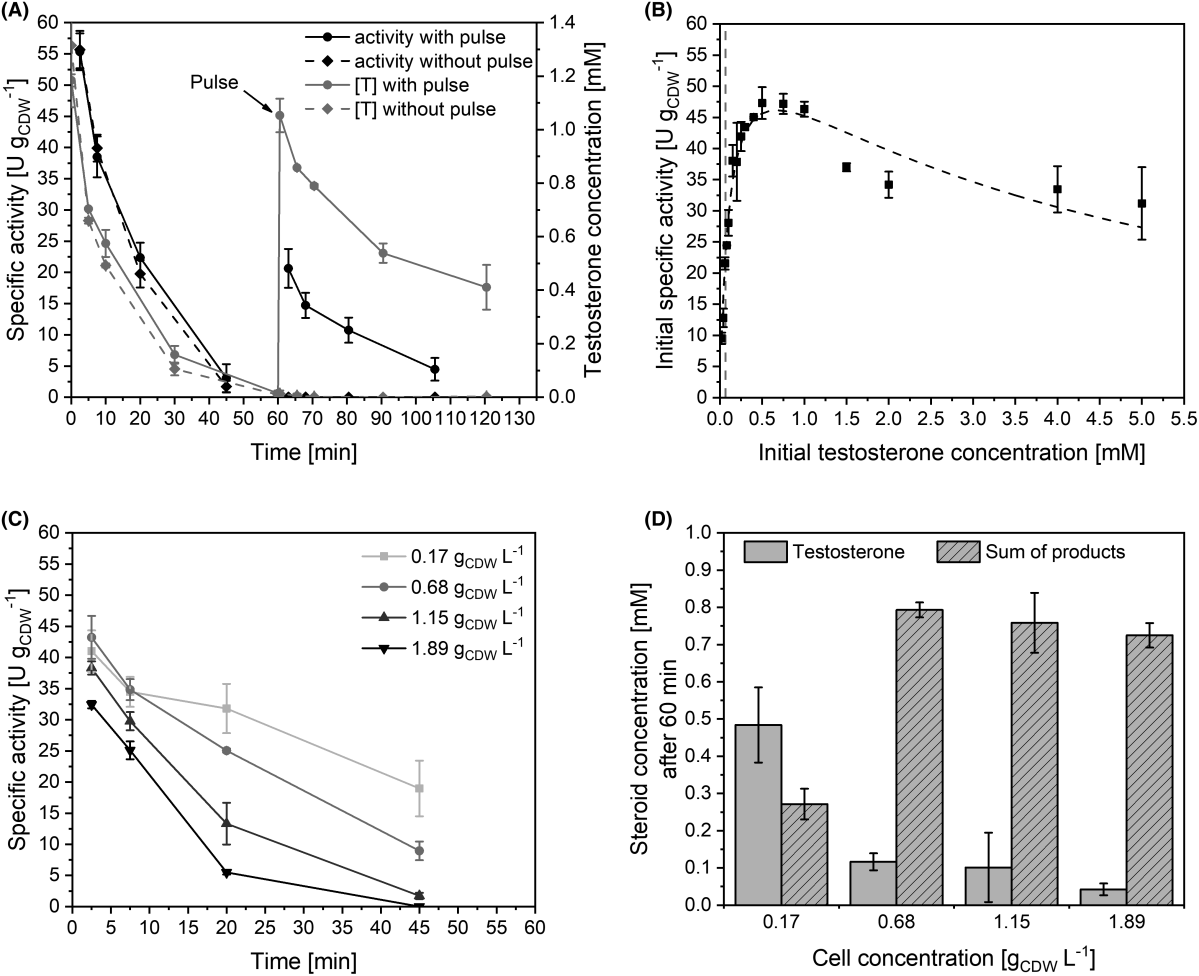
Substrate availability evaluation for testosterone hydroxylation catalysed by resting *E. coli* BL21‐Gold(DE3) cells equipped with KSA14m and AlkL. M9 medium containing 0.5% (w/v) glucose was used for bacterial growth and heterologous protein synthesis. Resting cells were prepared 5 h after induction and employed in activity assays as described in the Materials and Methods section. (A) Substrate pulsing: Testosterone biotransformation time courses with and without testosterone pulsing (1 mM) 60 min after reaction start were compared. Specific activities and testosterone concentrations are shown as black and grey lines, respectively. (B) Whole‐cell biotransformation kinetics: Initial activities (5 min) as a function of increasing initial testosterone concentrations, which reached saturation at 0.066 mM (grey dashed line). Higher concentrations need to be considered partially dissolved. Low cell concentrations of 0.09 g_CDW_ L^−1^ were applied to avoid a severe decrease in testosterone concentration during the 5 min reaction time. The dashed curve corresponds to a fit according to Michaelis–Menten kinetics with substrate inhibition utilizing the OriginPro 2018 software. (C) Specific activity courses using different cell concentrations as indicated. (D) Steroid concentrations in biotransformation mixtures with varied cell concentrations after 60 min of reaction. Standard deviations were obtained from two biological replicates.

First, we investigated, if and to what extent the resting‐cell biotransformations were limited by the availability of respective substrates (testosterone, NADPH, O_2_). When testosterone was pulsed after 60 min (1 mM), the specific activity of the cells recovered, but only to 37% of the initial rate at the same testosterone concentration (Figure 3A). This indicates that testosterone limitation was not the only cause for the observed activity decrease.

Kinetic analyses revealed a Michaelis Menten‐type behaviour for testosterone hydroxylation catalysed by KSA14m and AlkL‐containing *E. coli* with maximal activity obtained with a testosterone concentration around 0.5 mM, a substrate uptake constant (K_S_) of 0.11 ± 0.02 mM, a V_max_ of 61.2 ± 3.9 U g_CDW_^−1^ and weak substrate inhibition (K_i_: 4.1 ± 0.9 mM, Figure 3B). Interestingly, these kinetics were obtained for a substrate with a very poor solubility in the aqueous biotransformation buffer containing 1% (v/v) DMSO (~66 μM (Bertelmann et al., 2022)). Oversaturation however seemed to be necessary to ensure high activities with the (re‐)dissolving of testosterone as a possible rate‐limiting factor. Thus, the actual K_S_ may be even lower than the determined apparent K_S_.

The effect of substrate availability was further investigated by varying the whole‐cell biocatalyst concentration and thus varying the absolute substrate consumption rate with the same initial testosterone concentration (1 mM). Thereby, low cell concentrations led to an increased availability of both testosterone and O_2_. Initial specific activities did however not significantly differ with biocatalyst concentrations ≤1.15 g_CDW_ L^−1^ (Figure 3C), indicating that both substrates were not limiting at the biotransformation start. In contrast, initial activities decreased by 15% at the highest cell concentration tested (1.89 g_CDW_ L^−1^), indicating a limitation by O_2_ and/or a limitation by the (re‐) dissolving of testosterone in the applied biotransformation setup. For all cell concentrations tested, specific activities declined over time (Figure 3C). The activity decrease observed with a low cell concentration of 0.17 g_CDW_ L^−1^ and around 0.5 mM testosterone still available after 60 min of biotransformation (Figure 3D) corroborates that factors other than testosterone or O_2_ availability significantly contribute to the observed activity decrease. Below the threshold of 1.15 g_CDW_ L^−1^, the slower activity decrease with decreasing biocatalyst concentrations, involving slower product accumulation, indicates that the hydroxylated products may affect biocatalyst performance. Beside product inhibition of the enzyme itself, cellular toxicity of the products may compromise biocatalyst functionality.

As a third substrate, steroid hydroxylation catalysed by BM3 variants involves stoichiometric consumption of NADPH, which continuously is regenerated via the glucose catabolism of the whole‐cell biocatalyst. It has been reported that intracellular NAD(P)H regeneration and transhydrogenase activity in resting (i.e. non‐growing) *E. coli* cells can readily supply NADPH to both cellular maintenance and oxygenase catalysis (Blank et al., 2008; Fuhrer et al., 2005; Meyer et al., 2006), as demonstrated for several NADPH‐dependent reactions (Walton & Stewart, 2004). External supply of 1 mM NADPH did not have any effect on the initial specific activity, nor the activity decrease compared to control reactions without NADPH (Figure S1). NADPH is however not expected to be taken up by living cells. Glucose remained available throughout all biotransformations presented in this study as confirmed by glucose test stripes, when biotransformations were stopped.

In summary, we successfully avoided any substrate limitation (testosterone, O_2_, glucose) enabling some stabilization of the high steroid hydroxylation activity, which, however, still showed a significant decrease over time. Thus, we set out to investigate toxicities and inhibitions as possible causes for the observed biocatalyst instability.

### Steroid and DMSO toxicity evaluation

Hydrophobic compounds are known to penetrate membranes of microbial cells causing toxic effects such as membrane swelling, permeabilization and finally integrity loss (Sikkema et al., 1995). This can affect metabolism‐dependent biocatalytic reactions such as NADPH‐dependent testosterone hydroxylation (Kadisch, Willrodt, et al., 2017). As quantitative growth monitoring via OD_450_ measurements or dry weight determination was not feasible at high steroid levels due to disturbance by dispersed solid steroids, growth effects were evaluated on a theoretical basis referring to literature findings and data. Cell lysis is predicted at membrane concentrations (c_M_) exceeding 300–400 mM (Kratzer et al., 2015). The concentration of a hydrophobic compound in biological membranes depends on its concentration in the aqueous phase (c_w_) and its partitioning between membrane and aqueous phase, that is its membrane‐water partition coefficient (*P*_M/W_) (de Bont, 1998). A correlation between the *P*_M/W_ and *P*_O/W_ (partition coefficient in an octanol–water system) has been reported (Sikkema et al., 1994):

(1) $$\log P_{M/W}=0.97\;\log P_{O/W}\;–\;0.64$$

**FIGURE 4:**
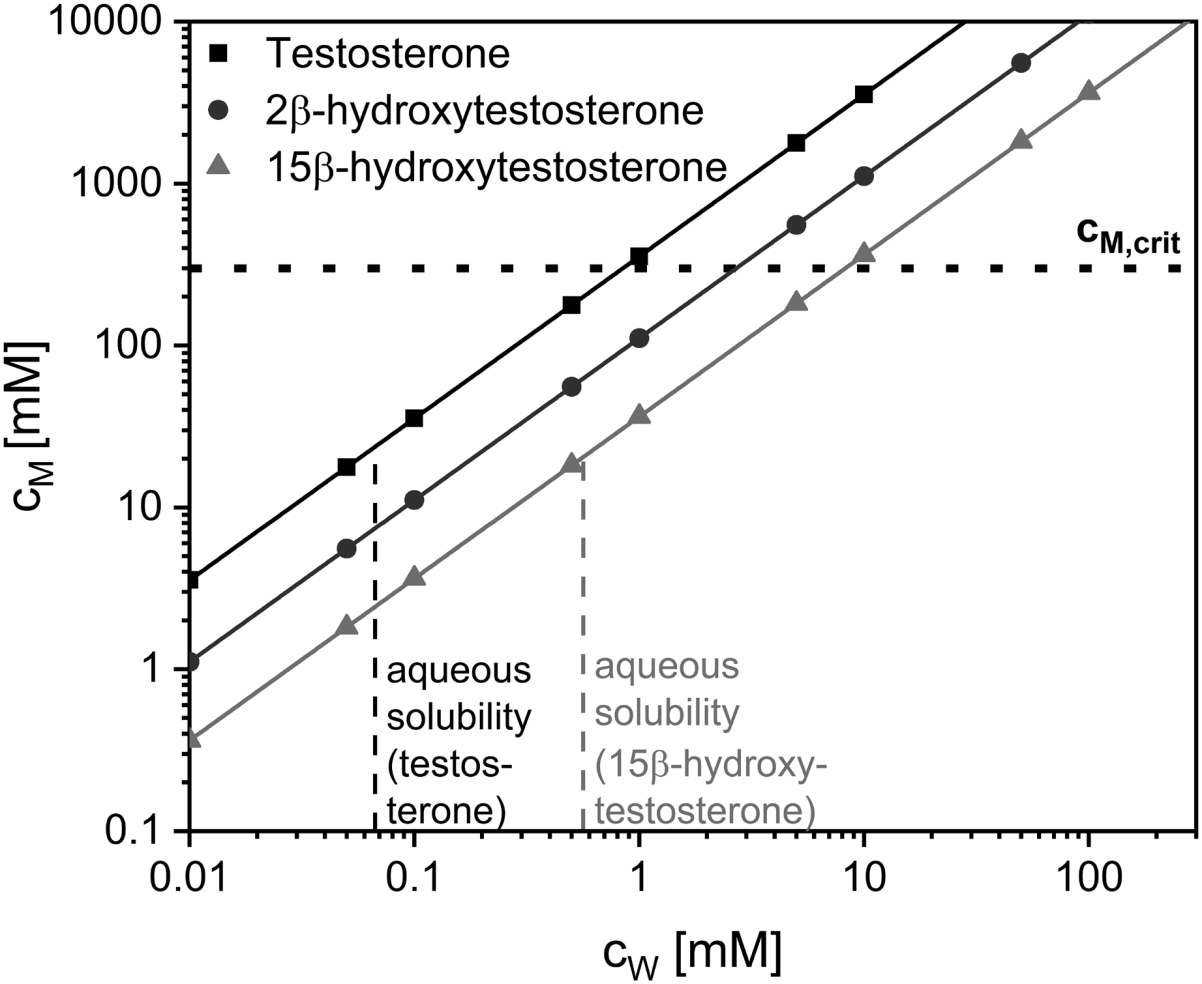
Toxicity evaluation for testosterone and its hydroxylated products: Theoretical correlation of membrane concentrations (c_M_) and aqueous concentrations (c_W_). Calculations were based on Equation 1 and computed log*P*_O/W_ values. Toxic effects are expected when the membrane concentration exceeds a threshold (c_m,crit_) of 300 mM (dotted line). Experimentally determined solubility limits of testosterone (66 μM) and 15β‐hydroxytestosterone (565 μM) in the aqueous biotransformation buffer containing 1% (v/v) DMSO are depicted as dashed lines.

Equation 1 enables predictions regarding the toxicity of hydrophobic compounds based on their log*P*_O/W_. Highly hydrophobic compounds with a log*P*_O/W_ > 4 are generally considered non‐toxic due to their low water solubility and the barrier function of the outer membrane (Laane et al., 1987), which however was relieved in the presence of AlkL. Organic solvents with a log*P*_O/W_ between 1 and 4 on the other hand easily reach a toxic c_M_ and generally are considered highly toxic for microbes. Computed log*P*_O/W_ values for testosterone and the products 2β‐ and 15β‐hydroxytestosterone amount to 3.3, 2.8 and 2.3, respectively (National Center for Biotechnology Information (2023)), being in the toxic range. Based on these log*P*_O/W_ values, critical membrane concentrations (c_M,crit_) of 300 mM are theoretically reached at aqueous concentrations of 0.8, 3 and 8 mM, respectively (Figure 4). Such aqueous concentrations are, however, not reached as they excel the respective solubilities experimentally determined for the applied reaction buffer (66 μM for testosterone and 565 μM for 15β‐hydroxytestosterone). Consequently, all steroids are not expected to significantly affect cell viability and functionality under the applied conditions. For the substrate testosterone, this is corroborated by the determined apparent substrate inhibition constant (K_i_) of 4.1 mM (Figure 3B). These estimations however have to be interpreted carefully since they rely on computed log*P*_O/W_ values and an empirically found correlation that is significantly influenced by external conditions such as temperature, medium salinity and, most importantly, the type of hydrophobic compound and possible interactions of dispersed solids with membranes (including their solubilization therein).

Due to the very low aqueous solubility of testosterone, stock solutions are usually prepared in water‐miscible organic solvents (Agematu et al., 2006; Brixius‐Anderko et al., 2015; Litzenburger & Bernhardt, 2017; Schmitz et al., 2014; Zehentgruber, Drǎgan, et al., 2010; Zehentgruber, Hannemann, et al., 2010) and added to the whole‐cell biocatalysts to initiate biotransformation. For the AlkL‐containing whole‐cell biocatalyst applied in this study, testosterone addition via DMSO allowed specific activities of ~45 U g_CDW_^−1^ as compared to 32.4 ± 1.4 U g_CDW_^−1^ upon surplus addition of testosterone without DMSO, rendering the co‐solvent strategy superior. However, toxic concentrations of co‐solvents can affect whole‐cell biocatalyst functionality (Kadisch, Willrodt, et al., 2017). Toxicity studies revealed that the used standard content of 1% (v/v) DMSO (log*P*_O/W_: −0.6 (National Center for Biotechnology Information, 2023)) did not have an effect on bacterial growth in M9 medium (Figure S2A), but higher DMSO levels reduced the specific growth rate (by 17% with 2.5% v/v) or even led to a linear growth behaviour (10% v/v). Respective effects on the initial specific activity were less prominent (Figure S2B). To conclude, the co‐solvent DMSO can be excluded as a main factor responsible for the observed activity decay, but long‐term effects remain to be studied under real process conditions.

### The main product 15β‐hydroxytestosterone inhibits testosterone hydroxylation, but is not the only cause for the observed activity decrease

**FIGURE 5:**
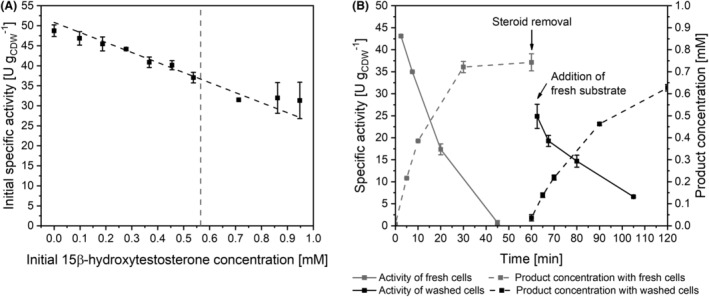
Influence of the main product 15β‐hydroxytestosterone on the catalytic performance of *E. coli* BL21‐Gold(DE3) cells harbouring KSA14m and AlkL. Bacterial growth and heterologous protein synthesis were conducted in M9 medium containing 0.5% (w/v) glucose. Resting cells were prepared 5 h after induction with 0.1 mM IPTG and applied in activity assays as described in the Materials and Methods section. (A) Influence of different 15β‐hydroxytestosterone concentrations on the initial specific testosterone hydroxylation activity (5 min). Linear fitting (black dashed line) was performed using the software OriginPro 2018. 15β‐hydroxytestosterone solubility in the assay buffer (565 μM) is shown as grey dashed line. (B) Reuse of whole‐cell biocatalysts in resting‐cell assays after steroid removal in fresh buffer. Specific activities (solid lines) and corresponding product concentrations (dashed lines) are depicted. Data points represent average values and standard deviations of two biological replicates.

Next, we tested a possible inhibitory effect of the main product of KSA14m, that is 15β‐hydroxytestosterone. Compared with the very low testosterone solubility in the biotransformation buffer (66 μM), the solubility of 15β‐hydroxytestosterone is 8.6 times higher (565 μM). This significantly increases its availability to the biocatalyst and may cause inhibitory effects. To test such effects, initial specific testosterone hydroxylation rates were determined upon exposure to different 15β‐hydroxytestosterone concentrations. The correlation did not follow a classic inhibition curve. Instead, the activity was reduced linearly with increasing initial product concentrations (Figure 5A). Linear fitting revealed a reduction by 25.1 ± 1.8 U g_CDW_^−1^ per mM of 15β‐hydroxytestosterone. Interestingly, concentrations above the solubility limit appeared to not further reduce the hydroxylation activity. We further examined, if the activity is irreversibly lost or can be recovered when formed product is removed from the cellular environment. For this purpose, resting‐cell biotransformations were performed for 60 min followed by whole‐cell biocatalyst harvesting via centrifugation, a washing step, and resuspension in fresh steroid‐free buffer. The absence of hydroxylated product was confirmed via HPLC analysis (Figure 5B). After re‐starting the reaction by addition of 1 mM testosterone, 58% of the initial hydroxylation activity were recovered. This is more than the activity recovery upon the relief of substrate limitation (37%, Figure 3A), indicating a partial reversibility of the activity loss. This product‐related difference in activity recovery indicated inhibition by 15β‐hydroxytestosterone, which, however, seems to be reversible to a certain degree. The limited recovery of hydroxylation activity and the difference in the correlation of activity decrease and 15β‐hydroxytestosterone concentration upon initial product addition (Figure 5A) and product accumulation during biotransformation (Figure 5B) indicate that product inhibition is one, but not the only effect responsible for the observed activity decrease.

Overall, compromising toxic effects are considered unlikely, because theoretical membrane concentrations of steroids did not reach critical levels due to their low aqueous solubilities and the co‐solvent DMSO did not cause any inhibition (Figure 4). Instead, a product‐related inhibition occurs. Thus, the biotransformation and its scale‐up to a gram scale can be expected to profit from in situ product removal. However, the extent of activity reduction observed for washed cells indicates that substrate‐ and product‐independent aspects contribute to the activity decrease.

### Low catalyst stability under reaction conditions

**FIGURE 6:**
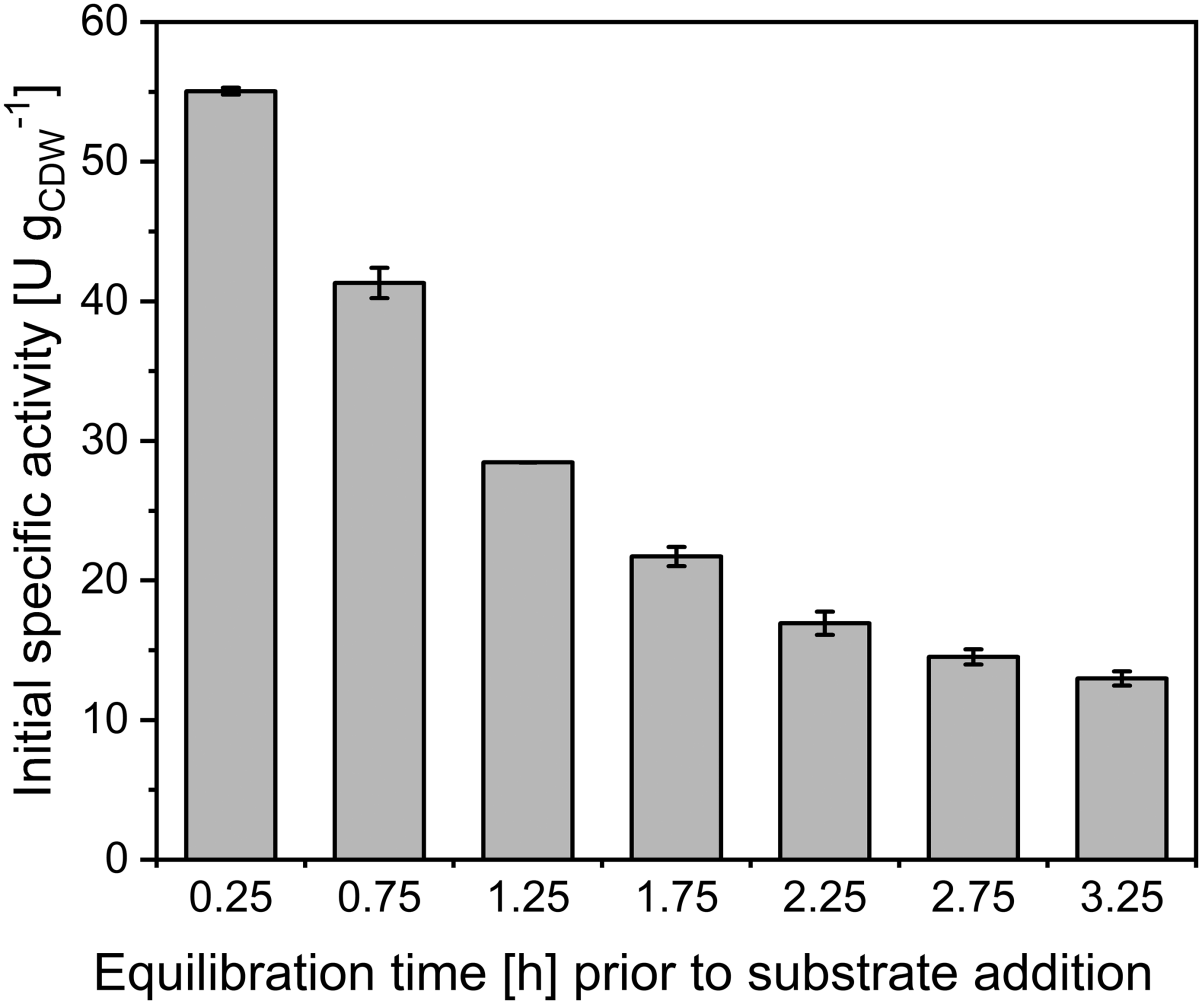
Biocatalyst stability under reaction conditions in the absence of testosterone. *E. coli* BL21(DE3)_pETM11‐ksa14m‐alkL cells were grown in M9 medium containing 0.5% (w/v) glucose and induced by addition of 0.1 mM IPTG. Resting cells were prepared 5 h after induction, incubated for different time periods under reaction conditions, and then provided with testosterone to initiate short‐term activity assays (5 min). See Materials and Methods section for experimental details. Bars represent average values and standard deviations of two biological replicates.

To investigate biotransformation‐independent whole‐cell biocatalyst stability, the normally applied equilibration time under reaction conditions prior to biotransformation start (0.25 h) was prolonged. Initial specific testosterone hydroxylation rates declined with extended pre‐incubation (Figure 6). The activity decreased to 50% of the initial rate after just 1 h prolonged pre‐incubation and appeared to even out at ca. 10–15 U g_CDW_^−1^. Obviously, the biocatalyst features a rather high biotransformation‐independent inherent instability.

### Induction kinetics corroborate inherent instability of the BM3 variant KSA14m

**FIGURE 7:**
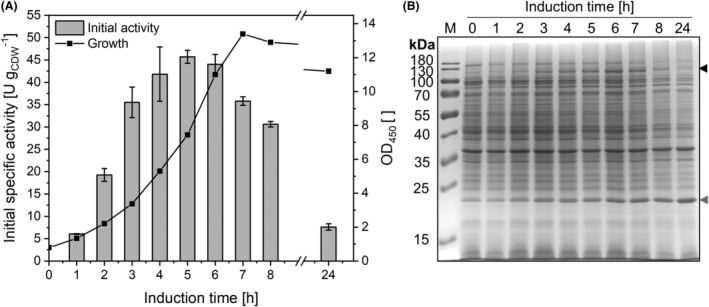
Induction kinetics of *E. coli* BL21‐Gold(DE3)_pETM11‐KSA14m‐alkL during batch growth in M9 medium with 0.5% (w/v) glucose at 30°C. (A) Specific testosterone hydroxylation activities (5 min, grey bars) as a function of the induction time during batch cultivation. Resting cells were obtained at indicated time points of growth (black line) and analysed in activity assays as described in the Materials and Methods section. Bars and data points represent average values and standard deviations of two biological replicates. (B) SDS‐PAGE analysis of cells harvested at different time points after induction. The sizes of KSA14m (119 kDa, black arrow) and AlkL (23 kDa, grey arrow) are indicated.

The applied reaction conditions have been used for many different *E. coli*‐based whole‐cell biotransformations and can generally be considered suitable to maintain basic metabolic functions and cell integrity (Cornelissen et al., 2013; Julsing, Kuhn, et al., 2012; Julsing, Schrewe, et al., 2012). To further investigate intracellular synthesis and stability of the BM3 variant KSA14m, induction kinetics were analysed. No product formation was detected before induction, after which specific testosterone hydroxylation rates increased steadily (Figure 7A) correlating to the increase in relative KSA14m levels (Figure 7B). Induction for 5 h led to the maximum activity and was considered optimal for expression. Upon glucose depletion (between 6 and 7 h after induction), both specific activity—as determined in glucose‐replenished buffer—and KSA14m level severely declined. This indicates that declining protein synthesis after glucose depletion could not keep up with the obviously prominent decay of the instable enzyme. This was in stark contrast to the observed levels of the outer membrane protein AlkL, which, being part of the same operon as the *ksa14m* gene, remained high in the stationary phase. These findings confirm KSA14m instability as a prominent reason for the observed whole‐cell activity decrease. Throughout biotransformation, relative KSA14m levels were stable for 1 h of biotransformation (Figure S3) despite very low activity remaining at this time point (Figure 2B), indicating that enzyme inactivation rather than degradation plays an initial role in the activity decrease. KSA14m was not detectable on SDS gels after 24 h of biotransformation, again in contrast to stable relative AlkL levels under reaction conditions (Figure S3), which further demonstrates the low stability of the BM3 variant.

### Influence of expression and reaction temperature

**FIGURE 8:**
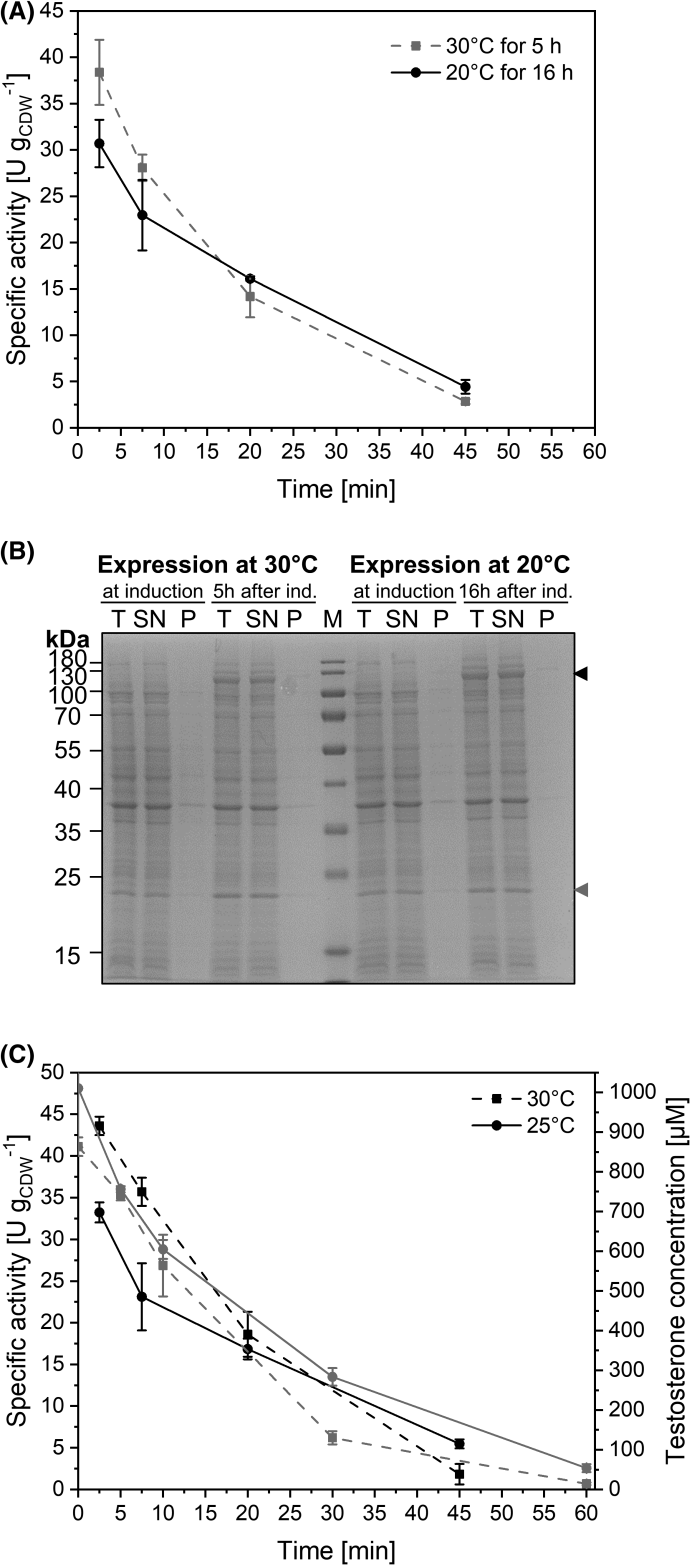
Effect of temperature variation on recombinant protein synthesis in and testosterone hydroxylation activity of *E. coli* BL21‐Gold(DE3)_pETM11‐ksa14m‐alkL. (A) Specific resting‐cell activities of cells obtained from M9 cultures incubated at 30°C for 5 h (grey dashed line) or 20°C for 16 h (black solid line) after induction with 0.1 mM IPTG. Resting cells were prepared and used for activity assays as described in the Materials and Methods section. (B) SDS‐PAGE analysis of recombinant cells with the sizes of KSA14m (119 kDa) and AlkL (23 kDa) indicated by black and grey arrows, respectively. Cells were sampled at the indicated time points. Fractions tested included T: total protein in crude cell extracts, SN: supernatant after centrifugation (soluble fraction) and P: resuspension of centrifugation‐derived pellet (insoluble fraction). (C) Specific activities (black lines) and testosterone concentrations (grey lines) for biotransformations performed at 25 and 30°C (solid and dashed lines, respectively). Resting cells were retrieved 5 h after induction from M9 cultures (30°C) supplemented with 0.5% (w/v) glucose. Data points represent average values and standard deviations of two biological replicates.

Functional production and stability of heterologous enzymes can well be temperature‐dependent. For example, high expression rates at high temperatures can lead to misfolding, which has been mitigated/avoided by lowering the expression temperature (Rosano & Ceccarelli, 2014). Thus, an expression temperature of 20°C was tested for heterologous KSA14m (and AlkL) synthesis. Induction was performed for 16 h (a time point found to be optimal for expression at 20°C, results not shown), and biotransformations were conducted at 30°C. This resulted in noticeably lower maximum specific activities and did not improve the kinetics of activity loss (Figure 8A). Comparison of soluble and insoluble protein fractions on SDS gels did not reveal any (detectable) inclusion body formation (Figure 8B). Lowering the biotransformation temperature to 25°C also did not lead to a more stable whole‐cell activity (Figure 8C), but, as it could be expected, resulted in a 24% lower initial specific activity compared to 30°C. Thus, variation of expression and reaction temperature did not lead to activity and stability improvements.

## DISCUSSION

### Critical aspects of CYP450‐based catalysis limit efficient steroid hydroxylation

Industrial implementation of CYP450‐catalysed steroid hydroxylations still faces a number of challenges related to the oxygenase systems applied, for example low turnover rates, instability, complexity and cofactor dependency. The recently reported highly active CYP450 BM3‐ and AlkL‐based whole‐cell biocatalysts (Bertelmann et al., 2022) were found to exhibit decreasing resting‐cell activities, indicating that instability issues may be critical regarding bioprocess implementation (Figure 2). This finding triggered us to study further bottlenecks in the 2β‐ and 15β‐monohydroxylation of testosterone catalysed by *E. coli*‐based whole‐cell biocatalysts carrying the BM3 variant KSA14m and AlkL (Figure 1). Inherent enzyme instability and product inhibition were identified as prominent causes for the observed decrease in whole‐cell activity. Limited substrate availability due to poor steroid solubility further compromised testosterone hydroxylation rates. In the following, we discuss the general significance of the identified limitations as well as strategies aiming at biotransformation stabilization and scale‐up.

### Substrate supply and product removal as crucial factors in steroid bioconversions

Beside their uptake across the outer *E. coli* membrane, which can be relieved via hydrophobic outer membrane porines, as shown in our recent study (Bertelmann et al., 2022), steroids and their hydroxylated derivatives typically have log*P*_O/W_ values between 1 and 4, which brings about the risk of whole‐cell biocatalyst toxification due to accumulation in the cellular membrane (Laane et al., 1987; Sikkema et al., 1995). Such toxification has been reported for various low logP_O/W_‐compounds, for example styrene oxide (Kuhn et al., 2013; Park et al., 2006), dodecanoic acid methyl ester (Kadisch, Julsing, et al., 2017), and cyclohexane (Bretschneider et al., 2021). Steroid toxicity may be further enhanced by high AlkL levels in the outer membrane and a consequential increase of intracellular substrate concentrations (Call et al., 2016; Julsing, Schrewe, et al., 2012; Kadisch, Julsing, et al., 2017). However, poor aqueous steroid solubility significantly reduces this risk as elaborated in the results section (Figure 4). On the contrary, with the steroidal substrate testosterone occurring as dispersed solids in aqueous media (Goetschel & Bar, 1992), accessibility to the biocatalyst constitutes another major challenge regarding biocatalytic steroid conversion. Steroids thus are mostly solubilized and supplied via water‐miscible organic co‐solvents, for example DMSO (Agematu et al., 2006; Bertelmann et al., 2022; Brixius‐Anderko et al., 2015), ethanol (Litzenburger & Bernhardt, 2017; Schmitz et al., 2014), methanol (Zehentgruber, Drǎgan, et al., 2010) or propan‐2‐ol (Zehentgruber, Hannemann, et al., 2010). In this study, however, maximum testosterone hydroxylation activities were only achieved upon oversaturation with substrate concentrations exceeding the solubility limit in the biotransformation mixture (Figure 3B). This observation indicates that direct dissolution of testosterone in the membrane may occur via AlkL and/or that dissolving of dispersed testosterone may become rate‐limiting. In both cases, scale‐up may benefit from an increase in aqueous testosterone concentration, which is hard to achieve as higher co‐solvent concentrations harm cell viability and functionality (Figure S2A).

Besides limited substrate availability, increasing aqueous product (15β‐hydroxytestosterone) concentrations were found to additionally compromise whole‐cell activities (Figure 5). In situ product removal (ISPR) thus may constitute a promising strategy to stabilize testosterone hydroxylation rates. The introduction of a second organic phase serving as substrate reservoir and product sink represents an interesting option (Bühler & Schmid, 2004; Willrodt et al., 2015; Witholt et al., 1990; Wubbolts et al., 1996). Beside water‐immiscible solvents, solid resins can be used for continuous substrate and product removal. Such concepts also allow to control product formation, for example by exploiting substrate and product partitioning to shift chemical equilibria, and facilitate downstream processing (DSP) (León et al., 1998; Salter & Kell, 1995; Woodley et al., 2008). Two‐liquid phase systems have been successfully applied for the oxygenase‐catalysed oxyfunctionalization of various hydrophobic substrates, inter alia, pseudocumene (Bühler et al., 2003), (+)‐valencene (Girhard et al., 2009), (*S*)‐limonene (Cornelissen et al., 2013) and styrene (Volmer et al., 2019). Also BM3 variants have been applied in biphasic systems, for example for the oxyfunctionalization of myristic acid using isolated enzyme (Maurer et al., 2005) and of α‐pinene with *E. coli*‐based whole‐cell systems (Schewe et al., 2009). *Yarrowia lipolytica*‐based whole‐cell bioconversion of progesterone with ethyl oleate as second phase constitutes an example for steroid hydroxylation in a biphasic system, which enabled a doubling of initial specific rates and prolonged product formation compared to monophasic systems (Braun et al., 2012).

Some bacterial strains possess adaptive solvent tolerance mechanisms (such as active solvent efflux and the alteration of membrane fluidity and surface properties), qualifying them as promising hosts for bioconversions involving toxic substrates/products or second phases (de Bont, 1998; Heipieper et al., 2007; Ramos et al., 2002). For example, solvent‐tolerant *Pseudomonas putida* MC2 has been used for toluene conversion to 3‐methyl catechol in a two‐liquid phase system with toxic octanol as carrier phase, allowing a 2.5‐fold increase in final product titre compared to the single aqueous phase approach (Hüsken et al., 2001). However, solvent tolerance mechanisms are typically accompanied by an increased energy demand possibly compromising specific activities and yields on energy source (Kuhn et al., 2012; Segura et al., 2005; Volmer et al., 2019).

The utility of solid resins for in situ substrate supply and product removal has been demonstrated for the production of 3‐phenyl‐catechol (Held et al., 1999), (*R*)‐(+)‐perillic acid (Mirata et al., 2009) and perillyl alcohol (Alonso‐Gutierrez et al., 2013). For the main product in this study, 15β‐hydroxytestosterone, Zehentgruber et al. demonstrated high extraction efficiency using Sepabeads SP207 (Zehentgruber, Hannemann, et al., 2010), rendering this resin promising for future ISPR and DSP attempts of hydroxylated testosterone derivatives.

Cyclodextrins and their derivatives play a major role in pharmaceutical research and development by acting as stabilizing and solubilizing carriers of hydrophobic drugs such as the steroids hydrocortisone and testosterone (Fenyvesi et al., 2019; Schwarz et al., 2017; Szejtli, 1988). This class of cyclic oligosaccharides also has been used to enhance microbial conversion rates of various steroids, for example cholesterol (Hesselink et al., 1989), androstenedione (Singer et al., 1991), 17α‐methyltestosterone (Druzhinina et al., 2008), cortisone acetate (Wang et al., 2009; Zhang et al., 2009) and phytosterol (Shen et al., 2012). Such positive effects derive from the capability of cyclodextrins to form water‐soluble non‐covalent inclusion complexes with hydrophobic compounds, which results in both the increase of steroid solubility and the decrease of potential toxic/inhibitory effects of steroids (Fenyvesi et al., 2019; Singer et al., 1991; Wang et al., 2009). Additionally and in contrast to many organic solvents used as co‐solvent or second phase, most cyclodextrin derivatives are biocompatible with both isolated enzymes and whole cells (Bar, 1989; Zhang et al., 2009). Previously, hydroxylation of several steroids was demonstrated up to millimolar scale using the derivative 2‐hydroxypropyl‐β‐cyclodextrin as solubilizing agent (Bracco et al., 2013), which thus is promising for more efficient steroid bioprocessing at high substrate concentrations.

### Tackling the inherent instability of BM3 variants

Oxygenases, including CYP450s, constitute a challenging enzyme class in terms of biocatalyst stability concerning both expression and activity. An issue frequently encountered is the uncoupling of NAD(P)H oxidation and substrate hydroxylation leading to partial oxygen reduction and concomitant ROS formation (Atkins & Sligar, 1987; Gorsky et al., 1984). This can be enforced by a non‐optimal fit in the binding site, for example of unnatural substrates (Lee, 1999; Loida & Sligar, 1993b), or by non‐oxidizable compounds, for example the biotransformation product (Suske et al., 1997). Furthermore, coupling efficiencies of CYP450s often become hampered when amino acid substitutions are introduced to modify substrate binding (Carmichael & Wong, 2001; Miles et al., 2000; Sowden et al., 2005). Compared to wildtype BM3 oxidizing natural substrates highly coupled to NADPH oxidation (93%–96% for long‐chain fatty acids) (Cryle et al., 2006; Noble et al., 1999), the single mutant F87A exhibits a poor coupling efficiency of 6.5% during testosterone hydroxylation (Kille et al., 2011). While the introduction of a smaller amino acid at this key active site residue vacated space for the bulkier non‐native substrate testosterone, the molecule's orientation in relation to the haem at the end of the substrate access channel likely is suboptimal for effective coupling. In general, uncoupling affects whole‐cell biotransformation efficiency by an increased oxygen demand, a waste of reduction equivalents and biocatalyst destabilization by ROS (Bernhardt & Urlacher, 2014; van Beilen et al., 2003). In this study, activity decrease inter alia was observed in the absence of biotransformation substrates/products (Figure 6, Figure 7A) and independently of expression and reaction temperature (Figure 8). The BM3 variant obviously decayed under expression conditions that enabled continuous gene expression, as can be concluded from the increasing AlkL levels with the respective gene being part of the same operon as *ksa14m* (Figure 7B). It has been reported that amino acid substitutions can enforce NAD(P)H oxidation in the absence of substrates (Whitehouse et al., 2010). Such oxidative stress can harm both protein function and cell physiology (Apel & Hirt, 2004; Cabiscol et al., 2000; Dickinson & Chang, 2011) and thus is disadvantageous for biocatalytic reactions relying on active metabolism (Kadisch, Willrodt, et al., 2017). Endogenous defence mechanisms of living cells against oxidative stress comprise one of the main reasons why living cells have emerged as preferred choice for oxygenase‐based biocatalysis (Leak et al., 2009; Schrewe et al., 2013; Woodley, 2006). However, ROS formation directly within the active centre can nevertheless promote active site destruction as reported for haem (Karuzina & Archakov, 1994a, 1994b). Adjusting oxygenase expression to a level that can be managed by cellular oxidative stress responses typically is crucial for whole‐cell biocatalyst stabilization (Kadisch, Willrodt, et al., 2017) and has been achieved by the variation of expression systems (Panke, Meyer, et al., 1999) and media (Bretschneider et al., 2021). Additionally, genomic integration of oxygenase genes has been shown to promote genetic stability, most likely profiting from low expression levels (Panke, de Lorenzo, et al., 1999). Upregulation of enzymes counteracting oxidative stress constitutes another promising approach for whole‐cell biocatalyst stabilization (Das et al., 2010). Also the application of growing instead of resting cells can convey superior biocatalyst stability due to efficient (re‐)synthesis of oxygenases (Schrewe et al., 2013) as well as more efficient handling of biocatalysis‐related stress like oxidative stress and product inhibition (Julsing, Kuhn, et al., 2012; Kuhn et al., 2010). Growing cells, however, often show lower activities and yields on energy source compared to resting cells (Julsing, Kuhn, et al., 2012) due to a trade‐off between demands for biomass synthesis and redox biocatalysis (Bühler et al., 2008).

Coupling efficiencies of CYP450s can also be improved by protein engineering to optimize the substrate fit in the binding pocket (Fasan, 2012; Loida & Sligar, 1993a). Previously, Arnold and coworkers achieved a complete remodelling of the active site of propane‐hydroxylating BM3 variants compared with the wildtype enzyme using a domain‐based engineering strategy (Fasan et al., 2008). This approach enabled an improvement of the coupling efficiency from 17% up to 98% and an 8‐fold increase of total turnover number, which ultimately translated in elevated and more stable in vivo activities during whole‐cell biotransformations (Fasan et al., 2007). A positive correlation of elevated total turnover numbers with better coupling efficiency has also been observed for BM3 variants capable of amorphadiene epoxidation (Dietrich et al., 2009), fluorene hydroxylation (Whitehouse et al., 2010) and propylbenzene hydroxylation (Whitehouse et al., 2010), qualifying the total turnover number as an excellent parameter to select for efficient coupling (Jung et al., 2011). The testosterone‐hydroxylating BM3 variants KSA2 and KSA14 evolved from F87A displayed higher activities (and better regioselectivity) coming along with improved coupling efficiencies of 30.0% and 45.8%, respectively (Kille et al., 2011). The KSA14m variant used in this study contains two more amino acid substitutions enabling higher testosterone conversion rates, but slightly reduced regioselectivity compared to KSA14 (Bertelmann et al., 2022). However, it remains unclear if these mutations influence the coupling efficiency as activity does not necessarily correlate with coupling efficiency (Whitehouse et al., 2012). The latter remains to be determined for KSA14m. Beside an improved coupling, the incorporation of (thermo)stabilizing mutations into BM3 prior to further engineering may help to generate variants with higher stability (Lewis et al., 2010). Finally, technical solutions such as ISPR may prevent product‐induced uncoupling.

## CONCLUSION

Living *E. coli* cells harbouring the BM3 variant KSA14m and the outer membrane protein AlkL showed testosterone hydroxylation at high rates, but poor stability. This work identified limited testosterone availability, product inhibition and inherent enzyme instability possibly involving uncoupling as critical factors. Inherent enzyme instability can be considered most critical and of general significance for efficient CYP450 catalysis. Engineering on the whole‐cell biocatalyst level, including enzyme and metabolic engineering, as well as on the reaction and process level, including in situ substrate supply and product removal, are considered necessary and promising to raise to full potential of the investigated highly active BM3‐ and AlkL‐based whole‐cell biocatalyst platform and enable unprecedented steroid bioprocessing efficiencies.

## AUTHOR CONTRIBUTIONS

**Carolin Bertelmann:** Conceptualization (equal); data curation (lead); formal analysis (lead); investigation (lead); methodology (lead); project administration (supporting); validation (equal); visualization (lead); writing – original draft (lead); writing – review and editing (equal). **Magdalena Mock:** Data curation (supporting); formal analysis (supporting); investigation (equal); methodology (supporting); supervision (supporting); writing – review and editing (supporting). **Andreas Schmid:** Funding acquisition (supporting); resources (equal); supervision (supporting); writing – review and editing (supporting). **Bruno Bühler:** Conceptualization (lead); funding acquisition (lead); investigation (equal); methodology (supporting); project administration (lead); resources (equal); supervision (lead); validation (equal); visualization (supporting); writing – original draft (supporting); writing – review and editing (lead).

## CONFLICT OF INTEREST STATEMENT

The authors declare that the research was conducted in the absence of any commercial or financial relationships that could be construed as a potential conflict of interest.

## Supporting information


Data S1.

## Data Availability

The original contributions presented in the study are included in the Supporting Information. Further inquiries can be directed to the corresponding author.
