# Supplementary material for: Efficiency aspects of regioselective testosterone hydroxylation with highly active CYP450‐based whole‐cell biocatalysts
Source: Microb Biotechnol. 2023 Nov 29;17(1):e14378. doi: 10.1111/1751-7915.14378 (PMC10832557; doi:10.1111/1751-7915.14378)
Supplement: Supplementary file 1 — Data S1. [file MBT2-17-e14378-s001.pdf]

Supporting information

Efficiency aspects of regioselective testosterone hydroxylation with highly active CYP450-based whole-cell biocatalysts

Carolin Bertelmann[1], Magdalena Mock[1,#], Andreas Schmid[1], Bruno Bühler[1,2*]

[1]Department of Solar Materials and [2]Department of Environmental Microbiology, Helmholtz Centre for Environmental Research GmbH - UFZ, Leipzig, Germany

[#]Present address: Department of Mechanical Engineering and Material Sciences, Georg Agricola University of Applied Sciences, Bochum, Germany

Correspondence: Bruno Bühler, Department of Environmental Microbiology, Helmholtz Centre for Environmental Research GmbH – UFZ, Permoserstraße 15, 04318 Leipzig, Germany

E-mail: bruno.buehler@ufz.de

Tel.: +49-341-235 46 87

Supplementary tables

Table S1. Amino acid substitutions present in the CYP450 BM3 variants used in this study. KSA1, KSA2, KSA3, and KSA14 originate from (Kille *et al.*, 2011), while KSA14m originates from (Bertelmann *et al.*, 2022).

Cytochrome P450 BM3 variant	Amino acid substitutions
KSA1	F87A / A330W
KSA2	R47I / T49I / Y51I / F87A
KSA3	R47I / T49I / Y51V / F87A
KSA14	R47Y / T49F / V78L / A82M / F87A
KSA14m	R47Y / T49F / V78L / A82M / F87A / K224E / V314I

Supplementary figures

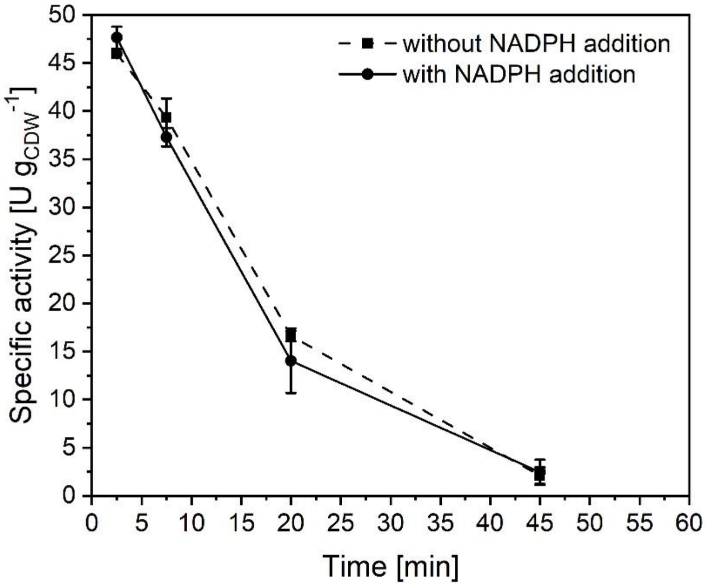

Figure S1. Specific testosterone hydroxylation activities of *E. coli* BL21-Gold(DE3) harboring pETM11 with the genes *ksa14m* and *alkL* in the absence or presence of 1 mM NADPH. Bacterial growth and heterologous protein synthesis were conducted in M9 medium containing 0.5% (w/v) glucose. Resting cells were prepared 5 h after induction with 0.1 mM IPTG and applied in activity assays as described in the Materials and Methods part. Data points represent average values and standard deviations of two biological replicates.

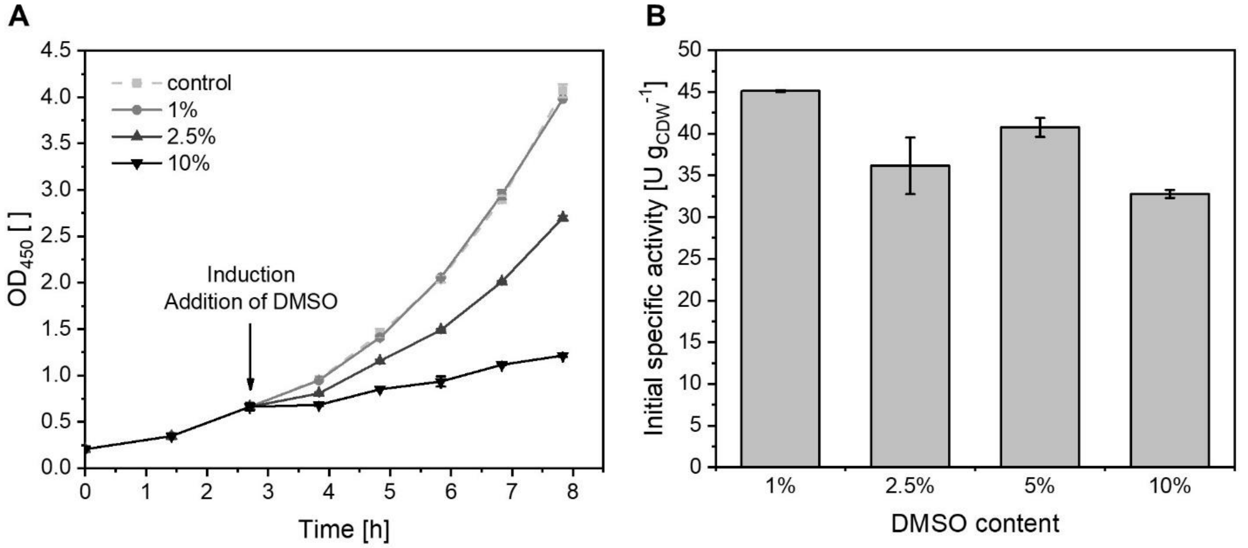

Figure S2. Effect of the co-solvent DMSO on growth and biocatalytic activity of *E. coli* cells carrying KSA14m and AlkL. M9 medium was supplemented with 0.5% (w/v) glucose and used for bacterial growth and heterologous protein synthesis by induction with 0.1 mM IPTG. For activity evaluations, resting cells were prepared 5 h after induction and used in assays as described in the Materials and Methods part. **(A)** Growth of induced cells in M9 medium after the addition of different DMSO concentrations compared to controls without DMSO. **(B)** Initial specific testosterone hydroxylation activities (5 min) in presence of different DMSO concentrations. Average values and standard deviations of two biological replicates are given.

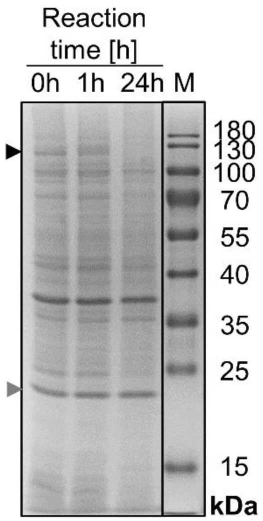

Figure S3. Stability of KSA14m and AlkL throughout the biotransformation as shown *via* SDS-PAGE analysis of cells harvested after 0, 1, and 24 h of biotransformation. KSA14m (119 kDa) and AlkL (23 kDa) are indicated by black and grey arrows, respectively. Bacterial growth and heterologous protein synthesis prior to the biotransformation were conducted in M9 medium containing 0.5% (w/v) glucose. Resting cells were prepared 5 h after induction with 0.1 mM IPTG and applied in activity assays as described in the Materials and Methods section.

References

Bertelmann, C., Mock, M., Koch, R., Schmid, A., and Bühler, B. (2022) Hydrophobic outer membrane pores boost testosterone hydroxylation by cytochrome P450 monooxygenase BM3 containing cells. *Front Catal* **2**: 887458.

Kille, S., Zilly, F.E., Acevedo, J.P., and Reetz, M.T. (2011) Regio- and stereoselectivity of P450-catalysed hydroxylation of steroids controlled by laboratory evolution. *Nat Chem* **3**: 738-743.
